# Decreased Extracellular Adenosine Levels Lead to Loss of Hypoxia-Induced Neuroprotection after Repeated Episodes of Exposure to Hypoxia

**DOI:** 10.1371/journal.pone.0057065

**Published:** 2013-02-21

**Authors:** Mei Cui, Xue Bai, Tianfu Li, Fangzhe Chen, Qiang Dong, Yanxin Zhao, Xueyuan Liu

**Affiliations:** 1 Department of Neurology, Shanghai Tongji University Affiliated Tenth People's Hospital, Shanghai, China; 2 Department of Neurology, Huashan hospital, Fudan University, Shanghai, China; 3 Epilepsy Center, Beijing Sanbo Brain Hospital, Capital Medical University, Beijing, China; Universidad de Castilla-La Mancha, Spain

## Abstract

Achieving a prolonged neuroprotective state following transient ischemic attacks (TIAs) is likely to effectively reduce the brain damage and neurological dysfunction associated with recurrent stroke. HPC is a phenomenon in which advanced exposure to mild hypoxia reduces the stroke volume produced by a subsequent TIA. However, this neuroprotection is not long-lasting, with the effects reaching a peak after 3 days. Therefore, in this study, we investigated the use of multiple episodes of hypoxic exposure at different time intervals to induce longer-term protection in a mouse stroke model. C57BL/6 mice were subjected to different hypoxic preconditioning protocols: a single episode of HPC or five identical episodes at intervals of 3 days (E3d HPC) or 6 days (E6d HPC). Three days after the last hypoxic exposure, temporary middle cerebral artery occlusion (MCAO) was induced. The effects of these HPC protocols on hypoxia-inducible factor (HIF) regulated gene mRNA expression were measured by quantitative PCR. Changes in extracellular adenosine concentrations, known to exert neuroprotective effects, were also measured using in vivo microdialysis and high pressure liquid chromatography (HPLC). Neuroprotection was provided by E6d HPC but not E3d HPC. HIF-regulated target gene expression increased significantly following all HPC protocols. However, E3d HPC significantly decreased extracellular adenosine and reduced cerebral blood flow in the ischemic region with upregulated expression of the adenosine transporter, equilibrative nucleoside transporter 1 (ENT1). An ENT1 inhibitor, propentofylline increased the cerebral blood flow and re-established neuroprotection in E3d HPC. Adenosine receptor specific antagonists showed that adenosine mainly through A1 receptor mediates HPC induced neuroprotection. Our data indicate that cooperation of HIF-regulated genes and extracellular adenosine is necessary for HPC-induced neuroprotection.

## Introduction

Transient ischemic attacks (TIAs) often warn of an impending stroke; it is known that 10.5% of patients who suffer from TIAs have a recurrent stroke within 90 days [1], [2]. Moreover, the majority of recurrent strokes occur within the first 30 days after a TIA, with more than half occurring in the first week [1], [3]. As such, achieving a prolonged neuroprotective state following TIA would effectively reduce the brain damage and neurological dysfunction associated with the expected recurrent stroke.

Hypoxic preconditioning (HPC) is a phenomenon known in which animals that are exposed to non-lethal, mild hypoxia have reduced stroke volume when subjected to an experimental stroke 1 to 3 days later [4], [5], [6]. For example, exposure to 8% O_2_ for 5 hours 3 days prior to middle cerebral artery occlusion (MCAO) significantly reduced stroke volume. This protective effect required 1 to 2 days to develop and was maximal at 3 days, disappearing within a week [4]. Thus, although this phenomenon represents a promising approach to the provision of advanced protection of the brain against an impending stroke, its protective effect is temporally restricted. Following TIA, it is impossible to predict the timing of a recurrent stroke, making a single HPC intervention clinically unfeasible.

In this study, we attempted to use multiple episodes of hypoxic exposure at different time intervals to induce longer-term protection from recurrent stroke. We demonstrated that exposure to hypoxia every 6 days (E6d HPC) decreased stroke volume, yet the protective effect was not long-lasting. Given that HPC-induced neuroprotection reached its maximal effect 3 days after hypoxic exposure, we tried hypoxic exposure every 3 days (E3d HPC); surprisingly, this procedure failed to protect the brain from ischemic insults. This intriguing data led us to study the underlying molecular changes leading to the loss of HPC-induced neuroprotection following frequent exposure to hypoxia. Hypoxia-inducible factors (HIF) have been identified as critical factors in mediating HPC-induced neuroprotection [4], [6], [7], [8].

HIF-1 is composed of two subunits: HIF-1α and HIF-1β. The HIF-1β subunit is constitutively expressed, while the expression level of HIF-1α is regulated by oxygen levels. Under normoxic conditions, the proline residues of HIF-1α are hydroxylated, which induces its association with the E3 ubiquitin ligase, von Hippel Lindau Factor, resulting in its degradation by the proteasome. Hypoxia blocks the activity of the HIF prolyl hydroxylases by reducing oxygen availability to the active centers of these enzymes. Through this mechanism HIF-1α levels are highly upregulated by HPC. HIF targets, including EPO and VEGF, have been shown to be protective during hypoxia preconditioning [4], [8].

Another potential target mediating the neuroprotective effects of HPC is adenosine and its receptors. Extracellular adenosine levels are low under normal conditions, but increase substantially in response to hypoxia. Adenosine is a potent endogenous neuromodulator with known neuroprotective properties. Adenosine acts on four G-protein coupled receptors: A1, A2A, A2B and A3 receptors. In the brain, the A1 subgroup is the most abundant and widespread of the adenosine receptors [9]. A1 receptors have the highest affinity for adenosine of all the subtypes [10]. A1 receptors are a central focus of the study of the neuroprotective role of adenosine in cerebral ischemia due to their high concentration and affinity for adenosine and known coupling to inhibitory G proteins.

In this study, we first examined the expression of HIF-regulated genes and extracellular adenosine levels in the mouse brain following different HPC protocols. By using different antagonists, we examined the roles of the key enzymes in adenosine metabolism and adenosine receptors in the loss of neuroprotection by frequent hypoxic exposure. Our data provide evidence that the cooperation of HIF-regulated genes and extracellular adenosine may be necessary for HPC-induced neuroprotection.

## Materials and Methods

### Animals

All experiments were conducted using male C57BL/6 mice (body weight: 18–25 g) housed at the Experimental Animal Center, Tongji University at a constant temperature and with a consistent light cycle (light from 07:00 to 18:00). This study was carried out in strict accordance with the recommendations in the Guide for the National Science Council of the Republic of China. The protocol was approved by the Animal Care and Use Committee of The Tenth People's Hospital of Shanghai (Permit number: 2011-0111). This study was also approved by the Science and Technology Commission of Shanghai Municipality (ID: SYXK 2007-0006).

### Hypoxic preconditioning

**Figure 1:**
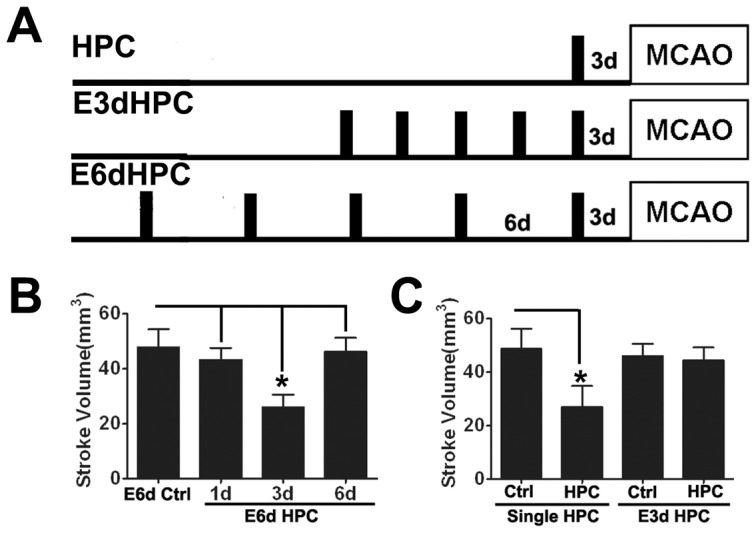
E3d HPC lead to the loss of HPC-induced neuroprotection. A schematic diagram shows the experimental protocols for HPC, E3dHPC and E6dHPC (A). Each black box represents an episode of hypoxia (8% O_2_ for 5 hours). Bar charts shows the stroke volume in mice following a single episode of HPC (C), E3d HPC (C) and E6d HPC (D). Stroke volume was evaluated 24 hours after MCAO. A significantly reduced stroke volume was induced by a single episode of HPC and E6d HPC, 3 days after the last exposure, although no effects were observed following E3d HPC. Data are shown as mean ± SD; *n* = 6–8 mice in each group; data analyzed by one-way ANOVA; * *P*<0.01 compared to the control.

For HPC, animals were transferred into an airtight, transparent chamber with a 3,000 mL volume, maintained at 21°C. A total gas flow (92% N2, 8% O2) of 400 mL/min of was established with the use of calibrated flow meters (Cole Parmer, Shanghai China), and partial oxygen pressure was measured intermittently by means of a polarographic electrode (Licox PO2, GMS, Keil, Germany). Animals were randomly assigned to different HPC or control groups. For each hypoxic exposure group, mice were exposed to either a single episode of HPC for 5 hours at 8% O2, or five episodes with the same exposure parameters at time intervals of 3 days (E3d HPC) or 6 days (E6d HPC; Fig. 1A). For all the controls to hypoxic exposure, mice were handled in the same manner, but were only exposed to room air. During HPC, animals had free access to food and water.

### Drug treatment

#### Adenosine uptake inhibitor treatment

The selective equilibrative nucleoside transporter inhibitor propentofylline (PPF) was purchased from Sigma-Aldrich (St. Louis, MO, USA) and dissolved in saline. Mice were randomly assigned to one of four groups: Control (exposed to room air), E3d HPC, E3d HPC +10 mg/kg PPF and E3d HPC+20 mg/kg PPF. In each group, mice received saline or PPF (intraperitioneally) 30 minutes before the start of MCAO or collection of microdialysis samples.

#### Adenosine receptors antagonists treatment

The selective adenosine A1 receptor antagonist, 8-cyclopentyl-1,3-dipropylxanthine (DPCPX), and the selective A2a receptor antagonist, 8-(3-chlorostyryl)caffeine (CSC), were purchased from Sigma-Aldrich. Drugs were dissolved in dimethyl sulfoxide (DMSO). Mice were randomly assigned to DMSO-treated, DPCPX treated or CSC treated groups. In each group, mice received DMSO, 0.1 mg/kg DPCPX or 1 mg/kg CSC (intraperitioneally) 30 minutes before the start of MCAO.

### Temporary middle cerebral artery occlusion (MCAO) stroke model, the measurement of regional cerebral blood flow and evaluation of neurological deficits

Three days after the last hypoxic exposure, mice were anesthetized with an intraperitoneal injection of ketamine and xylazine (65 mg/kg and 6 mg/kg, respectively) and placed in a stereotaxic frame (Kopf, Tujunga, CA, USA). The temperature of animals was maintained at 37°C using a heating pad and feedback control system (FHC, Bowdoin, ME, USA). A laser Doppler probe was fixed in place on the skull (5 mm lateral and 2 mm posterior to the bregma). A coated filament was placed into the external carotid artery lumen and advanced to the middle cerebral artery (MCA), with concurrent recording of laser Doppler cerebral blood flow to ensure that the cerebral blood flow decreased to below 25% of baseline. After 45 to 50 minutes, the filament was removed and the external carotid artery was permanently ligated. The temporary common carotid artery (CCA) ligation was then released and the Doppler probe was removed.

Infarct size was determined 24 hours or 72 hours later by staining the brain with 2,3,5-triphenyltetrazolium chloride (TTC). Only those mice in which a reduction in cerebral blood flow below 25% of the baseline was achieved, with a return to >80% of the baseline following filament removal, were included in data analysis. To account for the effects of edema, stroke volumes were calculated indirectly [11]. All mice meeting the above criteria were included in the analysis.

Neurological score was determined in each mouse 72 hours after MCAO, according to the following graded scoring system: 0 = no deficit; 1 = forelimb weakness and torso turning to the ipsilateral side when held by tail; 2 = circling to the affected side; 3 = unable to bear weight on affected side; and 4 = no spontaneous locomotor activity or barrel rolling [12].

Investigators who performed MCAO models, evaluation of infarct volumes and neurological scales were blinded to all the HPC protocols and drug treatments.

### Blood pressure, blood oxygen saturation, pH and blood cell counting

In a subset of animals, blood pressure, blood oxygen saturation, pH and blood cell counts were monitored via placement of a catheter in the femoral artery. Briefly, after being separated from the femoral vein and nerve, the femoral artery was ligated distally. The proximal end was clamped temporarily, and a plastic catheter was placed into the proximal femoral artery. A pressure monitor was used to measure the blood pressure throughout the operation. Blood samples were collected from the catheter to measure blood gases, as well as red and white blood cell counts.

### Quantitative PCR

Brain tissue was collected immediately after the last hypoxic exposure; tissue punches were collected from the cortex located in an area supplied by the MCA. This area was chosen because it is typically involved in the stroke volume after transient MCAO. Brain tissue was dounce homogenized, the RNA extracted, cDNA synthesized and quantitative PCR performed using previously described methodology [13]. Briefly, total RNA was collected using RNA Easy columns according to the manufacturer's recommendations (Qiagen, Valencia, CA, USA). Synthesis of cDNA was performed with SuperScript III (Invitrogen, Carlsbad, CA, USA) according to the manufacturer's recommendations. Quantitative PCR reactions contained 1 µM sense and 1 µM antisense oligonucleotides with SYBR green I master mix (Bio-Rad, Hercules, CA, USA). Primer sets (sense sequence, antisense sequence) for the following genes were: CD73 (5′-CAA ATC CCA CAC AAC CAC TG-3′, 5′-TGC TCA CTT GGT CAC AGG AC-3′); CD39 (5′-AGA CAG CTC CTG GGA ACA GA-3′, 5′- CAA CCA AGA GCA GGG AGA AG-3′); equilibrative nucleoside transporter-1 (ENT-1; 5′-AAC TCT CCA CCC ACC AAC AG-3′, 5′-AAC AGG CCA CAG GAA TGA AG-3′); erythropoietin (EPO; 5′-ACC CTG CTG CTT TTA CTC TC-3′, 5′-CAC AAC CCA TCG TGA CAT T-3′); vascular endothelial growth factor (VEGF; 5′-CTC ACT TCC AGA AAC ACG A-3′, 5′-GGG TGC TTT TGT AGA CTA TCA-3′); monocarboxylase transporter 4 (MCT4; 5′-AGG TCT GCC TGC TGT AAC ATT-3′, 5′-CCC TTT CTT CCT GCT GTA TGA-3′) and glucose transporter 1 (GLUT-1; 5′-TCA AAC ATG GAA CCA CCG CTAC-3′, 5′-GCC GAC AGA GAA GGA ACC AAT C-3′). Murine β-actin mRNA (sense primer: 5′-ACA TTGGCA TGG CTT TGT TT-3′; antisense primer, 5′-GTT TGC TCCAAC CAA CTG CT-3′) was amplified in identical reactions to control for the amount of starting template. All samples were measured in duplicate and standard curves of known concentrations of different genes were used. All mRNA expression level data is reported as a ratio to β-actin.

### Western blotting

Whole-cell lysates were collected from brain tissue located in an area supplied by the MCA. Briefly, radioimmunoprecipitation assay buffer with protease inhibitor (Sigma, St Louis, MO, USA) added at the recommended concentrations was used to lyse brain tissue. The brain tissue was then dounce homogenized and sonicated for 5 seconds, three times. The samples were then centrifuged at 13,000×*g* for 15 minutes and the protein-containing supernatant was collected. Proteins were separated by SDS-PAGE and then transferred to a nitrocellulose membrane before being placed in blocking buffer (5% non-fat dried milk, 0.1% BSA, 0.1% Tween, 34 mmol/L NaCl, Tris, pH 7.5) for 1 hour at room temperature. The membranes were subsequently incubated overnight at 4°C with the following primary antibodies: anti-CD39 (1∶1,000; Santa Cruz, Santa Cruz, CA, USA), anti-CD73 (1∶1,000; Santa Cruz, Santa Cruz, CA, USA), anti-ENT1 (1∶1,000; Chemicon, Billerica, MA, USA). anti-ADOA1R (1∶1000; Alpha Diagnostic International, San Antonio, TX, USA), anti-ADOA2aR (1∶500; Santa Cruz, Santa Cruz, CA, USA) or anti-tublin (1∶5,000; Sigma, St Louis, MO, USA). Secondary antibodies conjugated with horseradish peroxidase were used and immunoreactivity was visualized by chemiluminescence (SuperSignal Ultra, Pierce, Rockford, IL, USA). Bands of interest were analyzed and quantified using Scion Image.

### Measurement of extracellular adenosine

In vivo microdialysis was performed to measure extracellular adenosine levels as previously described [14]. Briefly, one day after the last hypoxic exposure, mice were prepared for MCAO as previously described. A locking intracerebral guide and stylet (Bioanalytical Systems, Inc, Mount Vernon, IN, USA) was then implanted in the ventrolateral right striatum using the following coordinates relative to the bregma: AP = +0.5 mm, L = −2.0 mm and H = −1.5 mm (from the brain surface). The external portion of the cannula was fixed to the skull with dental cement. Two days after guide cannula implantation, a microdialysis probe (2-mm membrane, Bioanalytical Systems, Inc, Mount Vernon, IN, USA) was inserted via the microdialysis guide cannula into the right striatum of freely moving mice. The probes were perfused with artificial cerebrospinal fluid (aCSF) or aCSF containing the CD73-specific inhibitor, α,β-methylene-adenosine diphosphate (AOPCP 1 mM; Sigma-Aldrich) or aCSF containing the ENT1-specific inhibitor, S-(4-nitrobenzyl)-6-thioinosine (NBTI 100 µM; Research Biochemicals, Natick, MA, USA) at a constant flow rate of 2 µL/min by means of a microperfusion pump (CMA/100 microinjection pump, Carnegie Medicine, Sweden). After at least a 2 hour equilibration period, the dialysates were collected every 30 minutes for 2 hours. Adenosine content in the samples was analyzed by high pressure liquid chromatography (HPLC) as previously described [15]. Briefly, samples were eluted on a reverse-phase column (Lichrospher-100, Merck, Philadelphia, PA, USA) of 250 mm×3 mm i.d. with a particle size of 5 µm, at a flow rate of 0.4 mL/min using a mobile phase containing 215 mM KH_2_PO_4_, 2.3 mM tetrabutylammonium hydrogen sulfate and 4% acetonitrile, adjusted to pH 6 with KOH. Signals were detected using a UV detector (D-UV 6000; Alltech, Deerfield, IL, USA) at 260 nm. Standard solutions were treated identically to samples. All peak areas were within the linear range of the standard curves. The adenosine values were extrapolated from the linear regression curve calculated on the basis of standard solutions. The extracellular adenosine levels are presented as the mean of four samples.

### Statistical analysis

All values are expressed as mean ± standard deviation (SD). Differences between means were analyzed using either one-way or two-way ANOVA followed by Newman-Keuls post-hoc testing for pair-wise comparison using SigmaStat v3.5. The null hypothesis was rejected when the *P*-value was <0.05.

## Results

### Multiple episodes of hypoxic exposure lead to loss of neuroprotection by hypoxic preconditioning

**Figure 2:**
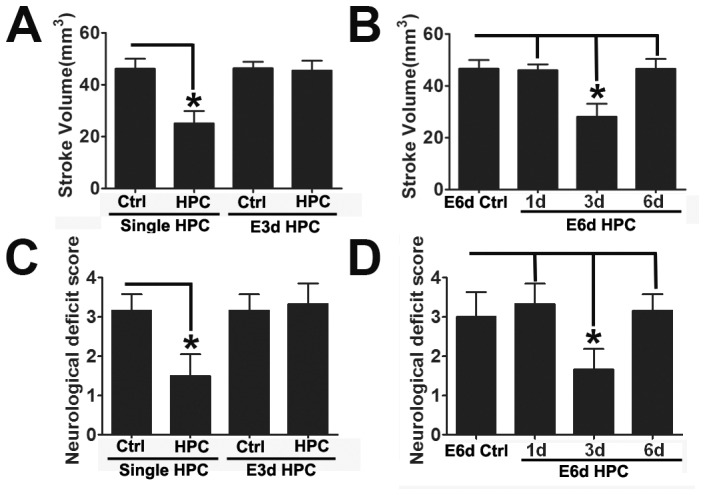
E3d HPC failed to provide protection against cerebral ischemia. Stroke volume was evaluated 72 hours after MCAO. E3d HPC failed to protect from ischemia 3 days after the last hypoxic exposure (A). Neurological deficits were also determined 72 hours after MCAO. Prominent improvements were observed 3 days after the last hypoxic exposure in the HPC and E6d HPC group (C, D). However, E3d HPC failed to ameliorate the neurological deficits induced by MCAO (C). Data are shown as mean ± SD; *n* = 6–8 mice in each group; data analyzed by one-way ANOVA; * *P*<0.01 compared to the control.

Consistent with previous studies, a single 5 hour exposure to 8% O_2_ 3 days prior to MCAO induced robust neuroprotection [4], as evidenced by a significantly reduced stroke volume (Fig. 1C, 2A) and also improved neurological deficits (Fig. 2C). For multiple episodes of hypoxic exposure, our E6d HPC protocol decreased stroke volume to about 25 mm^3^, yet the protective effect did not last for more than 6 days after the last hypoxic exposure (Fig. 1B, 2B).

Although different durations of HPC to induce neuroprotection have been reported, it is agreed that the protective effect reaches its peak 3 days after hypoxic exposure; our results are consistent with this view (Fig. 1C, 2A). Through the application of our E3d HPC protocol, we assumed that the HPC effect would overlap at its peak, providing a longer lasting neuroprotective effect. Surprisingly, E3d HPC failed to protect from ischemia even 3 days after the last hypoxic exposure (Fig. 1C, 2A, 2C).

As chronic intermittent hypoxia would change blood pressure and other physiological indexes, which may influence stroke volume, we measured blood oxygen saturation, blood pressure, pH, white blood cell count and hemoglobin in a subset of mice from each group. The results showed that none of our HPC protocols influenced these physiological indices (Table 1).

**Table 1:** Physiological parameters of mice treated with HPC.

	pH	PCO_2_ (mmHg)	PO_2_ (mmHg)	HCO_3_ (mmol/L)	WBC	HGB	Bp(mmHg)
**HPC Ctrl**	7.30±0.02	40.0±4.4	155.5±12.4	19.1±1.6	6.3±0.8	15.2±0.8	106±7.7
**HPC**	7.31±0.01	42.5±4.5	156.1±9.7	19.9±1.9	6.4±0.7	15.3±1.2	111±9.1
**E3d Ctrl**	7.30±0.01	39.4±5.0	157.6±10.2	19.6±1.9	5.9±0.5	15.3±0.9	108±4.8
**E3d HPC**	7.31±0.01	42.3±5.3	155.0±9.5	19.6±1.8	6.1±0.6	15.2±1.2	106±5.9
**E6d Ctrl**	7.31±0.01	41.2±4.9	151.0±9.8	19.7±2.6	6.4±1.0	15.4±1.0	106±7.5
**E6d HPC**	7.31±0.01	41.0±3.0	149.4±8.6	19.3±1.7	6.2±1.0	15.3±0.8	110±7.3

**BP,** blood pressure; **HGB**, hemoglobin; **WBC**, white blood cell count.

### Hypoxia-inducible factor targets remain highly upregulated following E3d HPC

**Figure 3:**
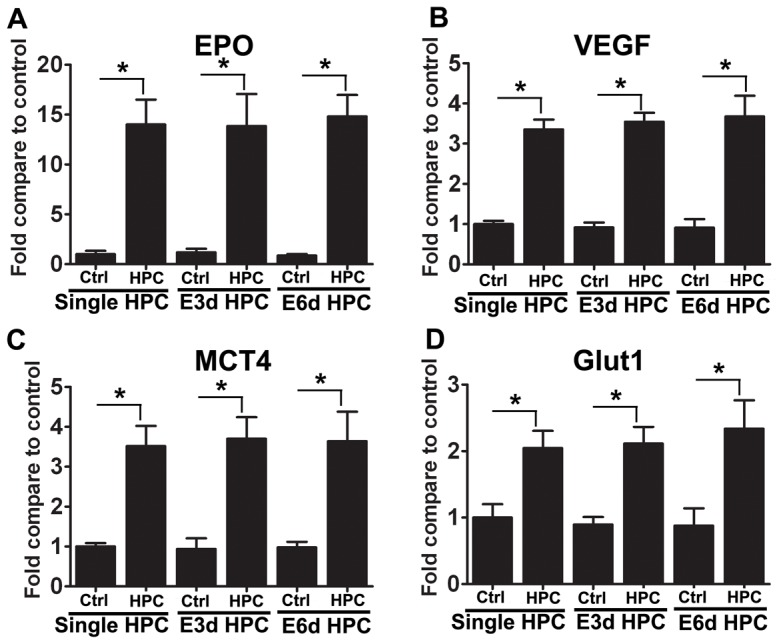
Hypoxia-inducible factor targets in the brain were still highly upregulated following E3d HPC. The mRNA expression levels of selected HIF targets in the region supplied by the MCA after the last hypoxic exposure are shown. A single episode of HPC, E3d HPC and E6d HPC significantly increased the expression of EPO (A), VEGF (B), MCT4 (C) and GLUT-1 (D). There was no significant difference between HPC groups. Data are shown as mean ± SD; *n* = 6 mice in each group; data analyzed by one-way ANOVA; * *P*<0.01 compared to the control.

Several molecules contribute to the protection mediated by HPC. It has been demonstrated that HIFs regulate genes contribute to HPC-induced neuroprotection, including EPO and VEGF [4], [7], [8], [16]. Thus, we examined the expression of HIF-1 targets in our experimental mice. Consistent with the neuroprotective effect, single HPC and E6d HPC induced the expression of the known HIF-1 targets, EPO, VEGF, GLUT-1 and MCT4 (Fig. 3A–D). In accordance with the loss of neuroprotection in E3d HPC mice, we assumed that HIF target genes would be decreased by this procedure; however, we also observed significant increases in the expression of EPO, VEGF, GLUT-1 and MCT4 in mice from the E3d HPC group (Fig. 3A–D). These results suggested that the upregulation of HIF target genes is not the only factor which explains HPC-induced neuroprotection against ischemic stroke.

### E3d HPC significantly decreased extracellular adenosine concentrations and reduced cerebral blood flow in the ischemic region of the brain

**Figure 4:**
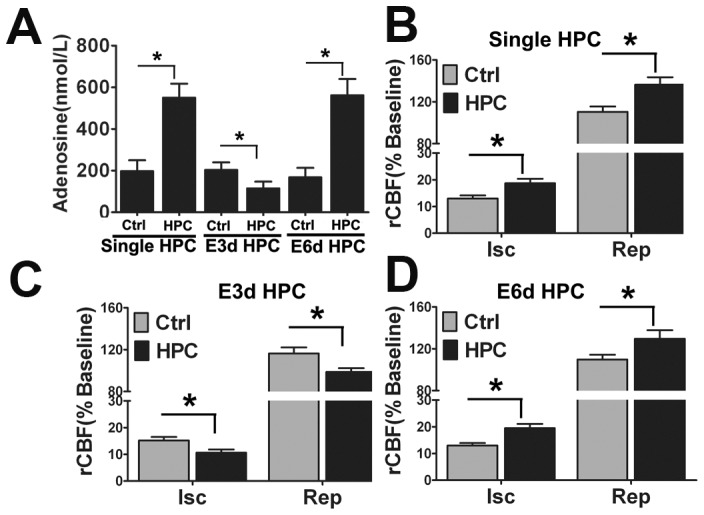
E3d HPC produced a dramatic reduction in the extracellular adenosine levels and cerebral blood flow in the ischemic region of the brain. Dialysates were collected every 30 min for 2 h followed by HPLC analysis of adenosine. The extracellular adenosine levels were presented as the mean of four samples. HPC and E6d HPC significantly increased extracellular adenosine levels in the striatum of mice, while E3d HPC decreased adenosine levels below those of the control (A). Regional CBF data are presented as a percentage of the baseline levels during both the ischemia and reperfusion stages. Single (B) and E6d HPC (D) significantly increased rCBF during both ischemia/reperfusion phases; however, rCBF in the ischemic region was significantly decreased by E3d HPC (C). Data are shown as mean ± SD; *n* = 6–8 mice in each group; data analyzed by one-way ANOVA in A and two-way ANOVA in B–D; * *P*<0.05 compared to the control.

In addition to upregulating HIF target genes, it is thought that HPC mediates protection of the brain through multiple mechanisms. Another important factor in this regard is extracellular adenosine, which has been strongly associated with HPC-induced neuroprotection, as well as cardioprotection and injury tolerance in general [17], [18]. ATP release into the extracellular space is increased during cellular stress such as inflammation and hypoxia [19], [20]. After being hydrolyzed by CD39 and CD73, which are ecto-enzymes located on the cell surface, the extracellular ATP is rapidly converted to adenosine [21]. In fact, after ischemic preconditioning treatment, is has been shown that extracellular adenosine levels are increased approximately 4-fold [22]. Therefore, the extracellular adenosine level was measured in experimental mice using in vivo microdialysis and HPLC. As expected, extracellular adenosine levels were increased to approximately 550 nmol/L after a single HPC episode, while in the E3d HPC group the extracellular adenosine concentration was reduced to approximately 115 nmol/L (Fig. 4A). This interesting finding suggested that the underlying molecular changes associated with the loss of HPC protection in the E3d HPC group may be the result of changes in adenosine metabolism.

Cerebral blood flow is critical for the survival of brain tissue in the ischemic region following stroke. Interventions that improve regional cerebral blood flow (rCBF), such as the use of statins to induce eNOS function, can ameliorate ischemic tissue damage [23]. Interestingly, adenosine is a potent cerebral vasodilator and has been proposed as a metabolic regulator of rCBF [24], [25], [26]. Given our observation of an association between reduced extracellular adenosine and loss of neuroprotection in E3d HPC mice, we examined the influence of different HPC protocols on rCBF. Our data indicate that a single episode of HPC and E6d HPC dramatically increased rCBF levels, consistent with a decrease in stroke volume and increases in extracellular adenosine concentrations (Fig. 4B and 4D). In contrast, E3d HPC led to a significant reduction in rCBF (Fig. 4C). This result supports the hypothesis that the loss of neuroprotection following E3d HPC may be associated with reduced rCBF, which may be secondary to a decline in extracellular adenosine levels.

### E3d HPC leads to a reduction in extracellular adenosine concentration by upregulation of ENT1 expression

**Figure 5:**
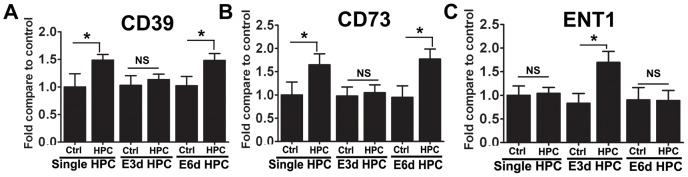
E3d HPC reversed the upregulation of CD39 and CD73 and induced ENT-1 mRNA expression. A single episode of HPC and E6d HPC significantly increased the mRNA expression levels of CD39 (A), CD73 (B) and had no effect on ENT-1 (C) expression. E3d HPC had the opposite effect, having no effect on CD39 (A) and CD73 (B) expression, but significantly increasing ENT-1 (C) expression. Data are shown as mean ± SD; *n* = 6 mice in each group; data analyzed by one-way ANOVA; * *P*<0.05 compared to the control.

**Figure 6:**
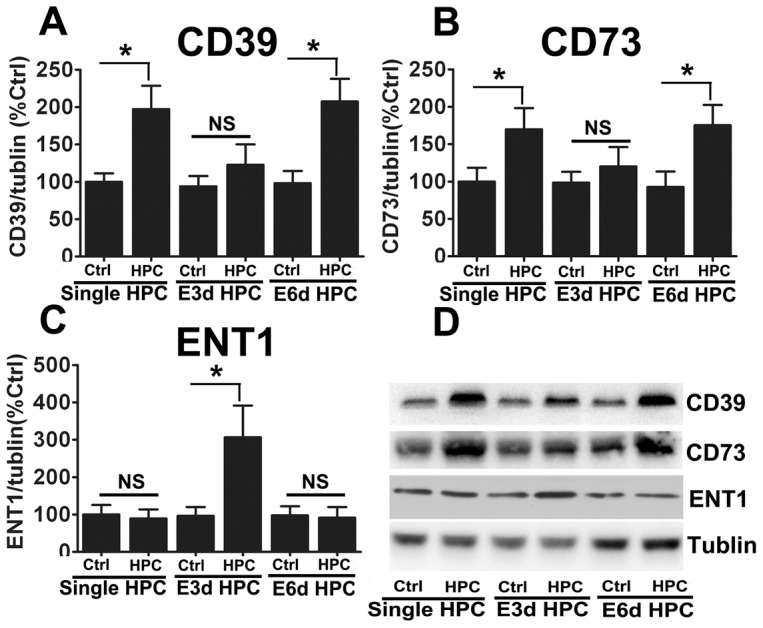
E3d HPC reversed the upregulation of CD39 and CD73 and induced ENT-1 protein expression. Consistent with our mRNA data, a single episode of HPC and E6d HPC significantly increased the protein expression levels of CD39 (A) and CD73 (B) and had no effect on ENT-1 (C) expression. E3d HPC had the opposite effect, having no effect on CD39 (A) and CD73 (B) expression, but significantly increasing ENT-1 (C) protein expression. Representative Western blots are shown in panel D. Data are shown as mean ± SD; *n* = 6 mice in each group; data analyzed by one-way ANOVA; * *P*<0.05 compared to the control.

Extracellular adenosine levels are regulated by a series of enzymes located on the cell surface, including CD39 and CD73, which facilitate the hydrolysis of ATP into adenosine. Although CD39 and CD73 are both promoted by HIF, they are also regulated by other promoters, such as SP1 and CREB [27], [28]. Because of their importance in regulating extracellular adenosine levels, we measured the expression levels of CD39 and CD73. Both CD39 and CD73 were highly increased at the mRNA (Fig. 5A and B) and protein levels (Fig. 6A and B, D) in the single HPC group; however, in the E3d HPC group, CD39 and CD73 mRNA (Fig. 5A and B) and protein levels were unchanged (Fig. 6A and B, D).

**Figure 7:**
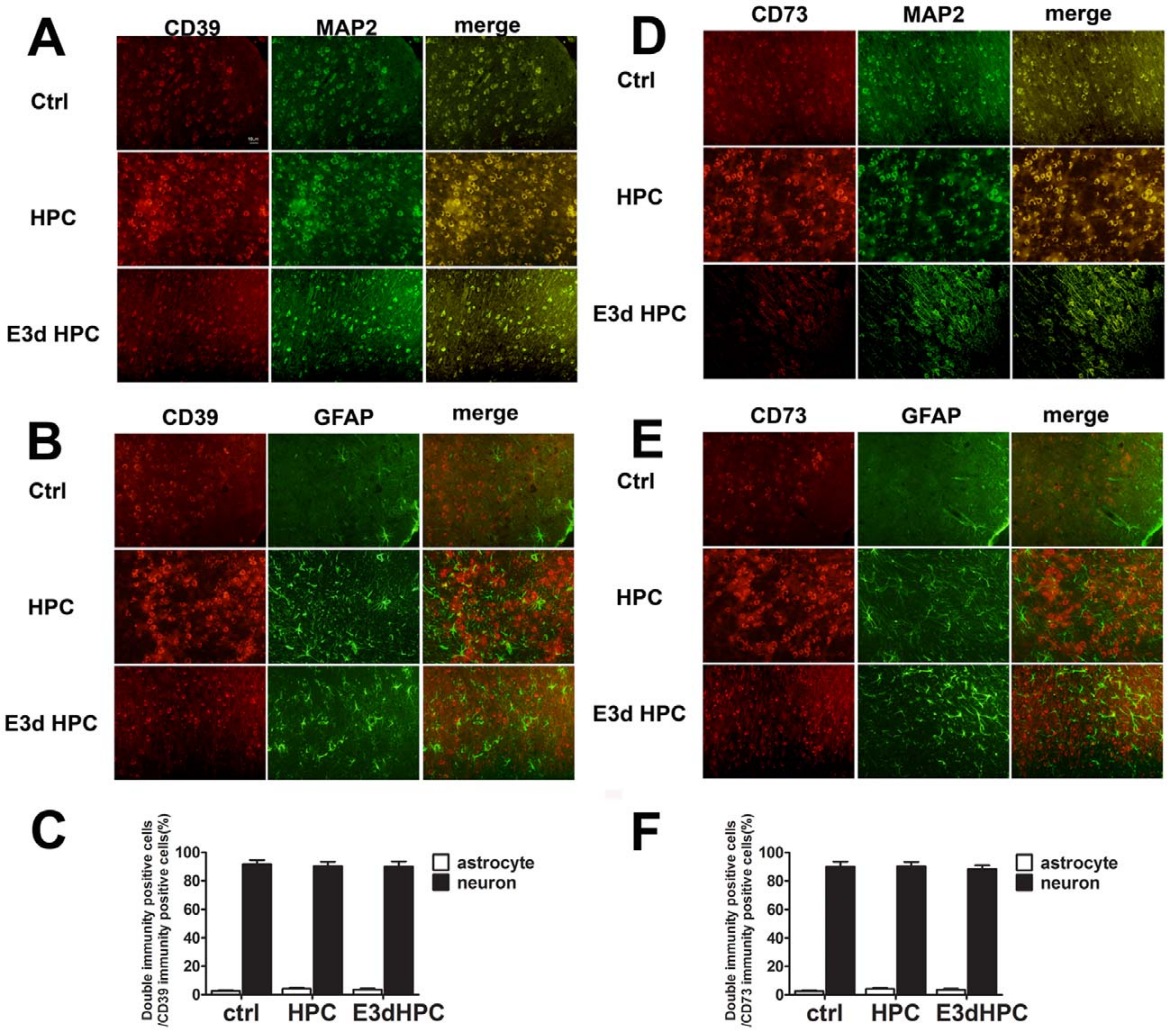
CD39 and CD73 were highly upregulated after HPC and were mainly distributed in neurons. Immunofluorescence assay was used to show the expression of CD39 (A, B, C) and CD73 (D, E, F) in neurons and astrocytes in different groups. Neuron and astrocyte cytoskeletal proteins, MAP-2 and GFAP, were labeled with green fluorescence, while CD39 or CD73 were labeled with red fluorescence. CD39 and CD73 were highly upregulated after HPC but not after E3d HPC (A, B, D, E). Quantitative analysis of receptor expression showed that both CD39 and CD73 were mainly localized in neurons (C, F). Data are shown as mean ± SD; *n* = 4 mice in each group.

The cellular distribution of CD39 and CD73 in neurons and astrocytes after different HPC protocols was determined by immunofluorescence assay and neurons and astrocytes were labeled by staining MAP-2 and GFAP, respectively. As shown in Figures 7A and D, CD39 and CD73 were expressed at low levels in normal mouse brains and both were mainly distributed in neurons, with less than 5 percent of CD39 and CD73 positive cells found to be distributed in astrocytes (Fig. 7B and E). Furthermore, both CD39 and CD73 expression significantly increased in neurons after HPC, while E3d HPC failed to influence CD39 and CD73 expression levels(Fig. 7A and D). More importantly, CD39 and CD73 were still mainly located in neurons after single or E3d HPC (Fig. 7C and F). This implied an essential role for neurons in production of extracellular adenosine.

**Figure 8:**
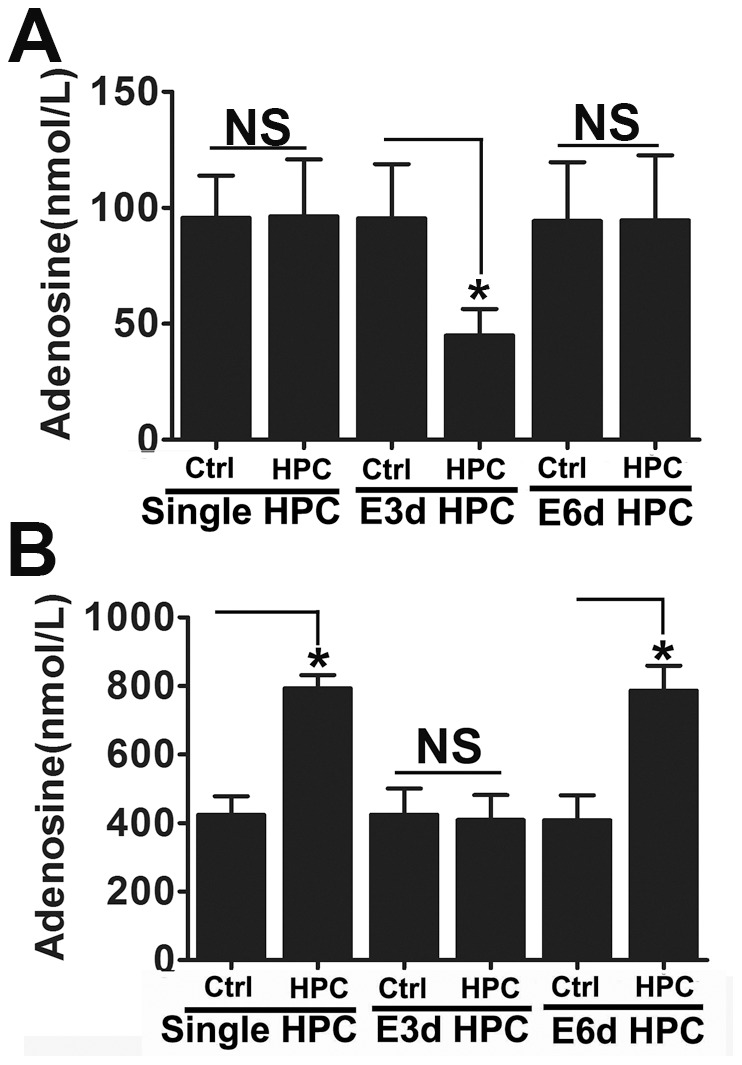
E3d HPC-induced reduction of extracellular adenosine levels were totally reversed by ENT1 inhibitor NBTI. After the last hypoxic exposure, animals received either CD73 inhibitor (AOPCP) or ENT1 inhibitor (NBTI) through the microdialysis infusate. AOPCP blocked the elevation of extracellular adenosine levels induced by HPC or E6d HPC (A), while NBTI totally reversed the E3d HPC-induced extracellular adenosine reduction (B). Data are shown as mean ± SD; *n* = 6 mice in each group; data analyzed by one-way ANOVA; * *P*<0.05 compared to the control.

Although ATP is degraded into AMP by multiple ectonucleotidases, only one ectoenzyme, CD73, degrades AMP into adenosine [29]. Based on this information, a CD73-specific inhibitor α,β-methylene-adenosine diphosphate (AOPCP), was used to block the production of extracellular adenosine. As shown in Figure 8A, after AOPCP treatment, HPC and E6d HPC lost the capacity to increase extracellular adenosine levels. This finding suggested that single HPC increased ATP metabolization into adenosine by upregulating CD73 expression.

ENT-1 is an adenosine transporter which also regulates adenosine levels in the extracellular space and transports excess extracellular adenosine into the cell [24], [30]. Although it is thought to be downregulated by HIF-1 [31], we did not observe any significant change in ENT-1 mRNA (Fig. 5C) or protein (Fig. 6C and D) expression in response to single HPC or E6d HPC. Interestingly, in the E3d HPC group, ENT-1 mRNA abundance (Fig. 5C) and protein levels (Fig. 6C and D) were significantly upregulated. This implied that E3d HPC leads to a reduction in extracellular adenosine concentrations by upregulation of ENT1 expression.

To test this hypothesis, the ENT1-specific inhibitor S-(4-nitrobenzyl)-6-thioinosine (NBTI) was infused into the striatum and extracellular adenosine levels were determined. After NBTI treatment, dialysate adenosine concentrations were upregulated under all conditions. Thus, the E3d HPC-induced extracellular adenosine reduction was totally reversed by NBTI. In contrast, NBTI had no effect on HPC and E3d HPC-induced upregulation of extracellular adenosine (Fig. 8B).

Taken together, it can be speculated that the decrease in CD39 and CD73 expression (which are key enzymes involved in extracellular adenosine production), and the increase in ENT-1 (which transports excess adenosine back into the cell) observed in mice exposed to E3d HPC may explain the decreased extracellular adenosine levels.

### Adenosine uptake inhibitor increased the cerebral blood flow and re-established the neuroprotection associated with E3d HPC

**Figure 9:**
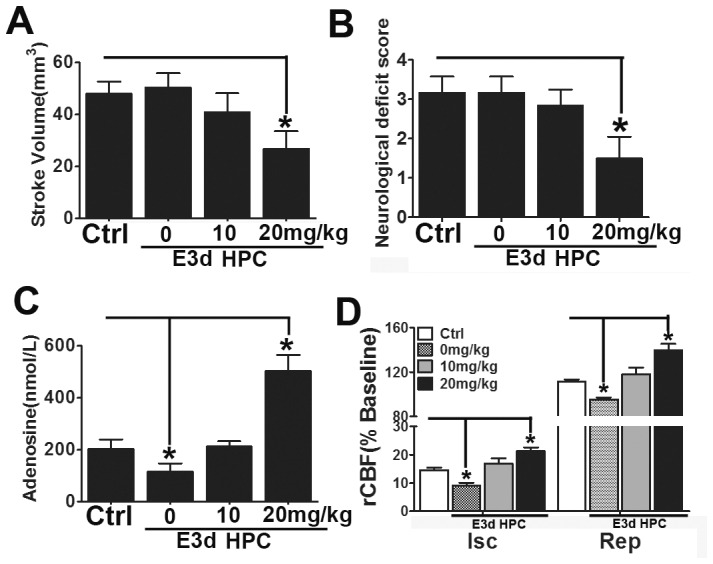
PPF re-established neuroprotection in E3d HPC mice and upregulated the regional cerebral blood flow. When delivered intraperitoneally, PPF markedly attenuated the infarct volume and neurological deficits after E3d HPC (A, B). Dialysate adenosine concentrations were also dramatically elevated (C). rCBF in the ischemic region was also significantly improved during both the ischemic and reperfusion stages (D). Data are shown as mean ± SD; *n* = 6 mice in each group; data analyzed by one-way ANOVA; *P<0.05 compared to the control.

Propentofylline (PPF) is a xanthine derivative with a broad spectrum of interesting pharmacological activities due to inhibition of adenosine re-uptake and selective inhibition of ENT1 [32]. In order to further verify the function of ENT1 on hypoxia preconditioning, different doses (10 mg/kg and 20 mg/kg) of PPF were administered intraperitoneally. PPF markedly attenuated the infarct volume and re-established the neuroprotective effects on E3d HPC mice in a dose-dependent manner (Fig. 9A and B). PPF completely abolished the E3d HPC-induced extracellular adenosine reduction (Fig. 9C) and dramatically increased the cerebral blood flow during both the ischemia and reperfusion stages(Fig. 9D). These findings further suggested that, in mice exposed to E3d HPC, the elevated expression level of ENT1 increased the transportation of extracellular adenosine back into cells. The reduction in extracellular adenosine levels and regional cerebral blood flow may lead to the loss of neuroprotection.

### Neuroprotection induced by hypoxic preconditioning is mainly mediated by adenosine A1 receptor

**Figure 10:**
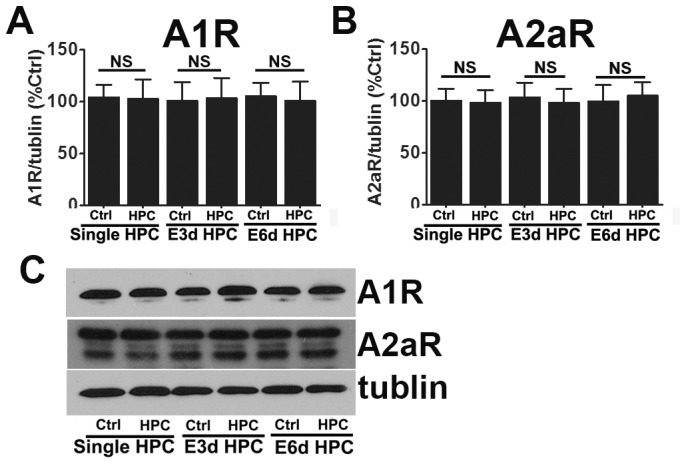
The expression of adenosine receptors in different hypoxia protocols. The protein expression level of adenosine A1 and A2a receptors were evaluated by Western blots. No changes in A1 and A2a receptor protein expression levels were observed. Western blots are shown in panel D. Data are shown as mean ± SD; *n* = 6 mice in each group; data analyzed by one-way ANOVA; * *P*<0.05 compared to the control.

Our results demonstrated that HPC and E6d HPC significantly increased extracellular adenosine levels in the striatum, while E3d HPC decreased adenosine levels below those of the control. We therefore examined the regulation of adenosine receptors in different hypoxia protocols. No obvious alternations in the expression of A1R and A2aR were observed (Fig. 10A–C).

The involvement of adenosine A1 and A2a receptors in regulation of neuroprotection after hypoxia preconditioning has been suggested [33], [34]. However, the exact function of these receptors remains a subject of debate [35], [36]. Therefore, in order to further explore the possible role of adenosine receptors in different HPC protocols, we investigated the consequences of both HPC and E3d HPC after treatment of mice with either an A1R or an A2a-specific antagonist.

**Figure 11:**
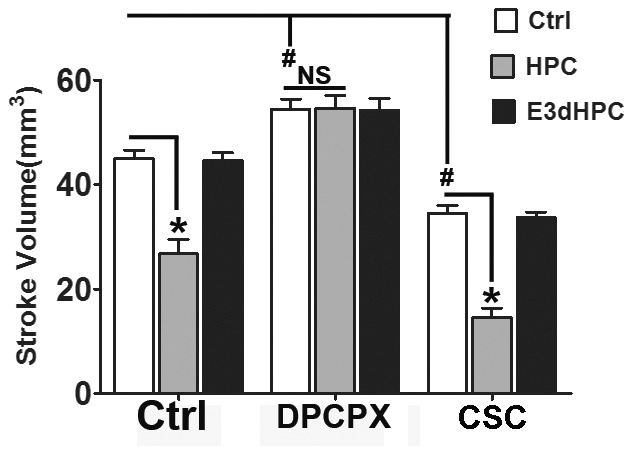
Adenosine A1 receptor antagonist DPCPX abolished the neuroprotective effects of HPC. The mice were treated with either the adenosine A1 receptor antagonist, DPCPX or the A2a receptor antagonist, CSC. DPCPX increased stroke volume and abolished the HPC-induced neuroprotection. CSC showed a detrimental effect on MCAO. However, the HPC induced neuroprotection was still observed. Stroke volume was evaluated 72 h after MCAO. Data are shown as mean ± SD; *n* = 6 mice in each group; data were analyzed by one-way ANOVA; * *P*<0.05 compared to control. ^#^ *P*<0.05 compared to the DMSO-treated control.

The neuroprotective effect of HPC was totally abolished by the adenosine A1 receptor antagonist DPCPX. Although the infarct volume was significantly reduced after A2a receptor antagonist CSC treatment in all groups, the neuroprotective effects of HPC remained (Fig. 11). These data indicate the neuroprotective effects of HPC result from upregulated extracellular adenosine, which acts primarily via the A1 receptor subtype.

## Discussion

HPC is a phenomenon in which advanced exposure to mild hypoxia reduces the stroke volume produced by a subsequent ischemic attack [4], [5], [6]. This phenomenon would be valuable in protecting the brain from stroke especially in patients with high risk of recurrent ischemic stroke. Although the beneficial effect of HPC has been established, the neuroprotection it provides does not last long. Commonly, the peak benefit occurs 1 to 3 days after HPC, with some variation among different protocols and the ischemic animal models employed [37], [38], [39]. Because the neuroprotective effect is transient, we employed different hypoxic protocols trying to induce longer-term protective effects before ischemic stroke. It has been reported that 3 days after hypoxic exposure, the neuroprotective function of HPC reaches its peak, yet this effect is lost by the sixth day [7], [38]. Our E6d HPC protocol was designed to provide sufficient time for recovery between hypoxic episodes. As expected, E6d HPC provided neuroprotection from an ischemic attack 3 days after the last exposure; however, the protective effect did not last longer than 6 days. Subsequently, we investigated our E3d HPC protocol based on the hypothesis that the peak neuroprotection of the last hypoxic exposure would be reinforced by subsequent exposure to hypoxia, thus sustaining the neuroprotective effect beyond 6 days. To our surprise, E3d HPC resulted in the loss of the protective function of HPC. This provoked our interest in exploring the underlying molecular changes responsible for the loss of neuroprotection.

The HIFs (HIF-1 and HIF-2) are transcription factors that orchestrate the molecular response to hypoxia. During hypoxia, the degradation of HIFs is blocked, leading to accumulation of these factors and upregulated transcriptional activity. HIFs targets, including EPO and VEGF, have been shown to be neuroprotective against acute cerebral damage, and have also been implicated in mediating the neuroprotection afforded by HPC [4], [8], [40], [41]. For example, EPO, which is expressed in the central nervous system, has been shown to have potent neuroprotective properties both in vivo [42], [43], [44] and in vitro [44], [45], [46].

Given the importance of EPO and VEGF in mediating the neuroprotective effects of HPC, we speculated that a decrease in the expression of theses HIF-regulated genes could underlie the loss of protection in our E3d HPC mice. Surprisingly, as in the single HPC and E6d HPC groups, we still observed elevated expression of HIF targets in E3d HPC mice. Specifically, the expression level of EPO was elevated (approximately 10-fold) after E3d HPC. This finding is consistent with previous reports of upregulated EPO mRNA and protein levels following single episode HPC [47], [48], [49]. Although there is solid evidence that exogenously applied EPO is neuroprotective both in vitro [44], [45], [46] and in vivo [42], [43], [44], the effect of endogenous EPO in neuroprotection is still debatable. For example, in transgenic mice overexpressing human EPO, infarct volumes tended to be smaller although this effect did not reach statistical significance [50]. In the present study, we observed a discrepancy in HIF target upregulation and loss of neuroprotection in the E3d group. This finding supports the hypothesis that HPC may induce the production of other potentially neuroprotective agents within the brain. This is consistent with our observation of increased HIF target gene expression in the absence of neuroprotection in E3d HPC mice. Taken together, this suggests that HIF target gene upregulation is insufficient to explain HPC-induced neuroprotection, and that other neuroprotective mechanisms must be involved.

One such factor that may also be involved in HPC-induced neuroprotection is extracellular adenosine. Adenosine is a purine nucleoside which acts via four subtypes (A1, A2a, A2b, A3) of G-protein-coupled cell surface receptors to restore homeostasis by increasing blood supply and decreasing energy demand. Adenosine increases blood flow through vasodilation of pre-existing vasculature and by stimulating angiogenesis [51], [52]. Adenosine has also been reported to activate presynaptic-A1 receptors, reduce glutamate release and to reduce activation of NMDA receptors. These synaptic inhibitory actions of adenosine exert a powerful neuroprotective effect during hypoxic and ischemic events [53].

The extracellular concentration of adenosine is controlled by the balance of its production and degradation through enzymes and by transmembrane transport processes. Several of these control mechanisms have been shown to be involved in HPC-induced protection. For example, in a myocardial infarct mouse model, the loss of CD39 increased infarct sizes and abolished cardioprotection by ischemia preconditioning [54]. Moreover, increased cerebral infarct volumes and reduced post-ischemic perfusion were also demonstrated in CD39 knock-out mice [55]. Interestingly, CD73, which hydrolyzes AMP to adenosine, was also shown to be necessary for cardioprotection by ischemic preconditioning [56]. Our data showed a failure to upregulate CD39 and CD73 in our E3d HPC mice, even though other HIF target genes were still highly upregulated. Although HIF-1 also acts as an important transcriptional regulator of CD73 and CD39 [57], other factors such as CREB (cAMP response element-binding) are also involved. Interestingly, it is known that the activation of HIF can recruit CBP which binds to and coactivates CREB, thus it is plausible that, under our hypoxic conditions, the repetitive activation of HIF would recruit endogenous CBP, and the coactivation of CBP to CREB would be reduced.

ENT1 is thought to be present on all cell types of the brain, including barrier endothelial and epithelial cells, neurons and glia [58], [59]. ENT1 mediates cellular influx or efflux of adenosine, with the direction of movement dependent upon the relative intra- and extracellular concentrations of adenosine. A recent study showed that during ischemic events, neuronal ENT1 activity leads to increased cellular uptake of adenosine, this uptake then decreases adenosine A1 receptor signaling and decreases the neuroprotective effects of adenosine [24]. Our data demonstrated that following E3d HPC, the expression of ENT1 was significantly increased.

The upregulated ENT1 expression together with the failure to elevate CD39 and CD73 may account for the decreased adenosine level in the E3d group. To test this hypothesis, we analyzed the influence of specific CD73 and ENT1 antagonists on extracellular adenosine levels in our different hypoxic models. Although the CD73 antagonist suppressed the upregulation of adenosine levels in the single HPC and E6d HPC groups, it did not change the decreased adenosine levels observed in the E3d group. While ENT1 inhibition totally reversed this pattern, thus suggesting an essential role for ENT1 in the decrease of extracellular adenosine levels by frequent hypoxic exposure. By using another ENT1 inhibitor PPF administered intraperitioneally, we went on to test the influence of ENT1 upregulation on stroke volume in the E3d group. As expected, this inhibitor mediated a dose-dependent increased adenosine levels and decreased stroke volume.

The adenosine A1 receptor (A1R) has the highest affinity for adenosine of all the adenosine receptors [10]. A1 receptor is generally considered to be protective in the context of cerebral damage. For example, A1R antagonist significantly increased cell death in the CA1 region in a mouse model of global ischemia [34]. Furthermore, the neuroprotective effects against stroke associated with delayed preconditioning are attenuated by the administration of the selective A1 antagonist [60]. The A2a receptor is also widespread in the central nervous system and binds adenosine with high affinity. However, its role in cerebral ischemia is debatable. Despite evidence showing that the relatively specific A2a agonist, CGS 21680, reduces ischemic damage [61], [62], several relatively specific A2a antagonists have been found to reduce ischemic damage in animal models of global or permanent ischemia [36]. In particular, genomic knock-out of the A2a receptor showed attenuated stroke volume in a focal cerebral ischemic model [33]. Since we observed a dramatic change in extracellular adenosine levels associated with single and E3d HPC, we went on to evaluate the effects of these two receptors on ischemic outcome under these different conditions.

In accordance with the protective role of the A1 receptor, the A1 antagonist not only increased stroke volume but also reversed HPC-induced neuroprotection, which suggests a critical role for extracellular adenosine in HPC. In contrast, our data support a detrimental role for the A2a receptor in cerebral ischemia although the HPC-induced neuroprotection still existed in the A2a antagonist treated animals. These results suggest that extracellular adenosine, which acts mainly through the A1 receptor, is essential for HPC-induced neuroprotection.

Adenosine is a potent endogenous vasodilator considered to be involved in local blood flow regulation to various tissues. It is also known that ischemic preconditioning before temporary MCAO causes a significant improvement in rCBF [63], although this effect is not thought to be associated with increased angiogenesis stimulated by the preconditioning stimuli. In our present study, rCBF was increased after either a single HPC or E6d HPC intervention, while in the E3d HPC group with the reduced extracellular adenosine levels, rCBF was significantly decreased during the ischemic and reperfusion stage. The ENT1 antagonist mediated a dose-dependent increase in extracellular adenosine levels and reversed the decrease in rCBF and enlarged stroke volume following E3d HPC exposure. We interpret this data to indicate that attenuated extracellular adenosine concentrations in the E3d HPC group led to the decrease in rCBF, which may contribute to the loss of neuroprotection.

In summary, the main findings of our study demonstrate that frequent hypoxic exposure leads to the loss of neuroprotection associated with HPC. By upregulating the expression of ENT1, E3d HPC results in a decrease in the extracellular adenosine levels and the decline in rCBF which are essential for the loss of neuroprotection.
